# Identification of CYP2C9 and VKORC1 polymorphisms in Iranian patients who are under warfarin therapy

**Published:** 2015-10-01

**Authors:** Behzad Poopak, Saghar Rabieipoor, Nazila Safari, Emadedin Naraghi, Fatemeh Sheikhsofla, Gelareh Khosravipoor

**Affiliations:** 1 DCLS, PhD in Hematology, Islamic Azad University, Tehran Medical Sciences Branch, Tehran, Iran; 2 MSc in Biotechnology, Payvand Clinical and Specialty Laboratory, Tehran, Iran; 3 MSc in Molecular Oncology, Payvand Clinical and Specialty Laboratory, Tehran, Iran; 4 MSc in Bioscience, Payvand Clinical and Specialty Laboratory, Tehran, Iran; 5 MSc in Cell and Molecular Biology, Payvand Clinical and Specialty Laboratory, Tehran, Iran; 6 MD, Payvand Clinical and Specialty Laboratory, Tehran, Iran

**Keywords:** CYP2C9 and VKORC1 polymorphisms, Polymerase Change Reaction, Warfarin dose requirements

## Abstract

**Background:** Although catalytic properties of different genetic polymorphisms of VKORC1 and CYP2C9 products have been identified, there is limited study available regarding warfarin dose requirement in Iranian patient population. This study investigates the impact of these polymorphisms on 115 patients, referred to Payvand Clinical and Specialty Laboratory for determining the appropriate dose of warfarin. Results of the study may be applicable to individuals who are under warfarin therapy to avoid warfarin resistance or intolerance.

**Subjects and Methods:** PT-INR test was utilized as a screening method. Genotyping were performed for VKORC1 and CYP2C9 using PCR method. Statistical analyses including unpaired t-test or ANOVA and regression were done using SPSS.

**Results:** VKORC1 GA was the most common genotype of VKORC1 allele among the study samples, with a rate of 57.4%. In CYP2C9 variant, 20% and 14.8% of subjects carried CYP2C9*1/*2 and CYP2C9*1/*3 genotyping, respectively. By contrast, the WT *1/*1 genotype was more abundant and dominant. The high frequency of VKORC1 (_1639) GA genotype (57.4%), was significant versus for the rest of the cohort (42.6%). In addition, a significant relationship was found between CYP2C9*1 and drug dose (P>0.021).

**Conclusion:** In this study, samples were characterized by higher frequencies of CYP2C9*1 and VKORC1 G/A, determined as higher warfarin taking doses. The results showed a significant relationship of the VCORC1 and CYP2C9 polymorphisms with warfarin sensitivity and severe side effects. Estimating right doses of warfarin to prescribe can help to reduce the risk of over- or under-anticoagulation and subsequently, the risk of thromboembolism or bleeding.

## Introduction

 Warfarin (Coumadin) is commonly used as an oral therapeutic anticoagulant in patients either with heart valves implants, atrial fibrillation or a confirmed episode of thrombosis or thromboembolism.   ^1^^-^^3^  Applying an individual treatment with warfarin requires careful monitoring due to the drug narrow therapeutic range for each person and unwanted side effects of this medication.   ^2^^,^^4^^ ,^^5^  The effectiveness of anticoagulation is monitored by International Normalized Ratio (INR) which is an index of its therapeutic effect. Currently, the PT-INR test is widely used to determine the appropriate dose of warfarin with the objective of evaluating the external path coagulation and diagnosis of hereditary and non-genetic (acquisitive) coagulation deficiency factors, based on the trial and error methods.   3^,^^6^^,^^7^  Many factors have been reported to be correlated with warfarin dosage such as age, sex, body mass, race and diet.^8^^,^^9^^,^^10^ Nevertheless, genetic polymorphism in different ethnic populations plays a more critical role in warfarin dosing.   3^,^^11^^,^^12^  S-warfarin enantiomer is particularly metabolized by hepatic cytochrome P450 2C9 and contributes to 70% of the anticoagulant impression of warfarin. In contrast, R-warfarin is metabolized by a number of enzymes such as CYP3A4, CYP1A1 (5,988 bp) and CYP1A2 (7,759 bp).   3^,^^4^^,^^13^  Moreover, during blood coagulation, Vitamin K plays a critical role. It is an important compartment that is anabolised by a hepatocyte membrane genes product of Vitamin K epoxide reductase complex subunit 1 (VKORC1).   3^,^^11^^,^^12^  With regard to the role of CYP2C9 and VKORC1 genes, it can be stated that these two genes are more linked to variations in polymorphisms among patients and play an important role in determination of warfarin drug dosage applied for treatment in each particular patient.  4^,^^6^^,^^14^  CYP2C9 as a primary enzyme responsible for inactivating warfarin, has a variant allele (CYP2C9*3) which shows a plunge (80%) in enzymatic activity of CYP2C9. Moreover, sensitivity to warfarin increases significantly in patients with a variant of VKORC1 gene (VKORC1-1639 A), as a result of about 50% reduction in transcription of VKORCR1 gene. In summary, studies indicate that these variants reduce capacity to metabolize warfarin that is clinically concerned with warfarin bleeding risk and necessity of lower doses of warfarin.   ^7^^,^^11^^,^^12^  However, studies show that the Caucasians require higher doses compared to Asiatic, which correlates well with their higher VKORC1 -1639 GG and VKORC1 -1639 AA genotype frequencies, respectively. The frequency of CYP2C9 polymorphism has been studied and reported in Amerindian language population.   6^,^^7^^,^^14^  

Patients, who are homozygous for CYP2C9∗3 and VKORC1-1639 A, are likely to be significantly sensitive to warfarin and are expected to require low dosages of warfarin to achieve appropriate therapeutic results and reduce the risk of side 

effects.   ^11^^-^^12^  However, limited information is available on VKORC1 and CYP2C9 polymorphisms in Iranian population. To understand the influence of these polymorphisms, warfarin dosage should be precisely identified.   3^,^^4^^,^^14^  The present study is designed to find out the effect of CYP2C9 and VKORC1 polymorphisms on warfarin dose requirements in Iranian population. The results of the study may be used in identification of patients with warfarin-sensitivity, which is a demanding subject due to the growing trend of development of cardiovascular diseases and unwanted complications in the primary stages of drug dose-usage in Iranian population. Estimating right doses of warfarin to prescribe can help to reduce the risk of over- or under-anticoagulation and subsequently, the risk of thromboembolism or bleeding.

## SUBJECTS AND METHODS

**Human Subjects**

 115 subjects were selected from Iranian population, referred to Payvand Clinical and Specialty Laboratory. The patients were under warfarin therapy due to heart valves implants, atrial fibrillation, or a confirmed episode of thrombosis or thromboembolism, etc. Clinical information including age (in average 15 yrs), gender (47% female and 53% male) and mean daily warfarin dosage (3.9 mg) were collected. In addition, samples with a mean of 2 to 3 for INRs were designated for this research. The study was performed by Payvand Clinical and Specialty Laboratory experts. The indication of identification polymorphisms included: patients bleeding, unstable INR, or based on the recommendations for warfarin genotyping.^15^

**PT-INR test**

PT test was utilized to evaluate the extrinsic and common pathways of coagulation cascade for this research as a screening method. This test must have been performed within 24 hours after citrated sample collection. Samples were centrifuged for 15 min at 1500g in Universal Behdad centrifuge prior to PT-INT testing. The sample plasmas (50 µL) were tested for prothrombin time by an automated coagulometer (Stago, STAR4).

**DNA extraction and genotyping**

A whole blood sample volume of 5mL was collected from each patient in a K2 EDTA tube. The target genomic region, detailed before, was subjected for study and the modified primers were employed. DNA extraction was performed with Genomic DNA purification kit (Fermentas Co, Lot: 00145461). The DNA concentration and quality were evaluated by Bio Photometer. Samples with 1.7-2.0 purity (OD=100 ng) were applied for the study. The extracted DNA was then genotyped for VKORC1 and CYP2C9 polymorphisms, using a Food and Drug Administration (FDA)-approved kit (Aid GmbH, Lot: 26-108) by PCR reverse dot blots. Suspicious cases were approved by PCR–RFLP method.

PCR procedure was applied on 50 µL Master Mix for each VKORC1, CYP2C9*2 and CYP2C9*3 variations. PCR products were digested by the restriction enzymes and visualized with Silver Nitrate (AgNO_3_). The Master Mix compositions, primer sequences, PCR conditions and restriction enzymes used (Fermentas), are summarized in Table 1. The results of genotyping were reported as VKORC1 normal (G) and mutant type (A) and CYP2C9 normal (wild) type (*1) and its *2 and *3 polymorphisms. 

**Statistical analysis**

Most of analyses were carried out using statistical software SPSS 19th version. The effect of genotype, warfarin intake dose, and warfarin recommendation dose were assessed using unpaired t-test or ANOVA. Moreover, regression was calculated to find factors contributing to warfarin dosage followed by linear regression to model the relationships between dose and other variables such as age, measured. Results were shown as mean - SD unless stated otherwise. A P-value of <0.05 was taken statistically significant.

## Results

**Patient characteristics**

 This study was carried out between December, 2010 and October, 2013 on patients who were referred to Payvand Clinical and Specialty Laboratory for investigation of targeted polymorphism regarding warfarin intake dose.

**Table 1-A:** Master Mix compositions

	CYP2C9*2	CYP2C9*3	**VKORC1**
**Buffer PCR 10x**	5 µL	5 µL	5 µL
**dNTP**	1.5 µL	1.5 µL	1.5 µL
**MgCl**_2_	3 µL	2.5 µL	2.5 µL
**Primer F**	1.5 µL	1.5 µL	1.5 µL
**Primer R**	1.5 µL	1.5 µL	1.5 µL
**Taq DNA polymerase**	0.2 µL	0.2 µL	0.2 µL
**Template**	1 µL	1 µL	1 µL
**Distilled Water**	37.8 µL	36.8 µL	36.8 µL

**Table 1-B:** Primer sequences and base pairs for CYP2C9 and VKORC1 polymorphisms

**Gene ** **name**	**Primer sequence**	**Length**
**CYP2C** 9*2	**Forward:** 5’ TACAAATACAATGAAAATATCATG 3’ **Reverse:** 5’ CTAACAACCAGACTCATAATG 3’	690 bp
**CYP2C** 9*3	**Forward 1:** 5 ’AATAATAATATGCACGAGGTCCAGAGATGC 3’ **Forward 2:** 5’ AATAATAATATGCACGAGGTCCAGAGGTAC 3’ **Reverse:** 5’ GATACTATGAATTTGGGACTTC 3’	166 bp
**VKORC** **1**	**Forward: **5**’** GCCAGCAGGAGAGGGAAATA 3’ **Reverse: **5’ AGTTTGGACTACAGGTGCCT 3’	290 bp

**Table 1-C:** Restricted enzyme protocols

**Restriction ** **enzyme**	**Break point**	**Polymorphism**	**Base ** **pair**
**AvaII**	5’…G↓G CC…3’	1*	169 bp 521 bp
**AvaIII**	5’…ATGCA↓T…3’	1*	31 bp 135 bp
**Kpn1**	5’…GGTAC↓C…3’	3*	31 bp 135 bp
**Msp1**	5’…CC G↓G…3’	G	123 bp 167 bp

**Table 2:** Patient characteristics; referred cases consisted of patients with Ebstein anomaly, foot inflation, lack of left lung, single ventricle and warfarin resistance disorder

**Sex**	**(%) of patient**
Male	53
Female	47
**Age group**	**(%) of patient**
1-10 y	23.5
11-30 y	36.5
31-50 y	20
51-70 y	13
71-90 y	7
**Indication for anticoagulation with warfarin**	**(%) of patient**
**Clot**	3
**Bleeding**	6
**Heart Disorder**	9
**Vein thrombosis/pulmonary embolism**	22
**Pulmonary vascular resistance (PVR)**	41
**Other** ^†^	19
**Resulted PT INR**	**(%) of patient**
2.0-3.9	39
4.0-5.9	61
**Total no. of Patients**	**115 (100)**

**Figure 1:**
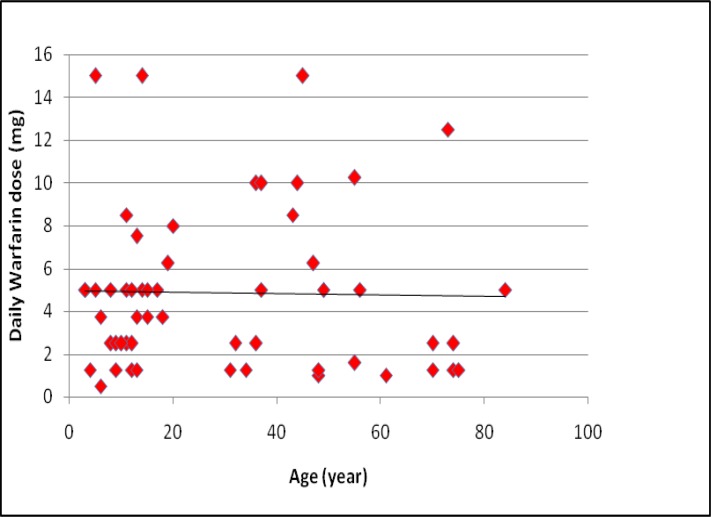
Relationship between daily warfarin dosage and age

A total of 115 patients with a median age of 15 years (range: 3-84 years) and a median duration of warfarin therapy of 24 months (range: 3-36 months) were analysed. The patient characterizations for anticoagulation therapy are illustrated in Table 2. Daily median warfarin dose was 3.4 mg (range: 0.5-15 mg). Figure 1 shows the relationship between warfarin daily dose and age. No significant difference was found between the groups concerning age (P> .0524, t-test).

**Genotyping results**

The frequencies of VKORC1 (GG, GA and AA), CYP2C9 (*1,*2 and *3) and also the combined polymorphisms of two genotypes of the study population are presented in Table 3. As it is shown in Table 3-A, in this study the VKORC1 GA polymorphisms was the most common genotype of VKORC1 allele among the sample population (57.4%). GG and AA genotypes had frequencies of 29.6% and 13%, respectively. In CYP2C9 variant, by contrast, the wild-type *1/*1 was the most abundant and dominant polymorphism (61.7%).

No significant difference was found between the groups concerning age (P> .0524, t-test).

About 20% and 14.8% of subjects carried CYP2C9*1/*2 and CYP2C9*1/*3 genotyping, respectively. Three individuals (3%) had CYP2C9*2/*3 genotype, while CYP2C9*3/*3 genotype was only detected in one of the samples. Similarly, frequencies of the combined polymorphisms of two genotypes are illustrated in Table 3-B. According to this table, 22 (19.1%) of patients had normal for both VKORC1 and CYP2C9. While 17 patients (14.8%) were heterozygote for both genotypes.

**Polymorphism relationship with warfarin dosage**

**Figure 2:**
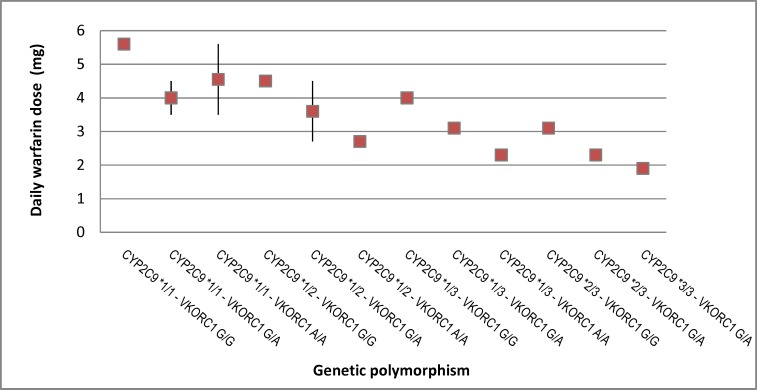
Relationship between genetic polymorphisms and recommended daily warfarin dose (in milligrams)

The daily mean warfarin dose requirement for optimal therapeutic in patients with wild-type CYP2C9 and VKORC1 (5.6 mg) was significantly higher than it in patients with the VKORC1 GA (4.5 mg) or AA (3.5 mg) genotypes. Also, the mean of recommended daily warfarin dose in patients with CYP2C9 *3/*3 and VKORC1 AA genotype (1.3 mg) was significantly lower than it in those with CYP2C9 *1/*2 with VKORC1 GG and GA (4.5 and 3.5 mg, respectively) and CYP2C9 *2/*3 with VKORC1 GG and GA (3 mg and 2.3 mg, respectively). In this study, the frequency of VKORC1 GA (57%) was significantly higher than it in VKORC1 GG and AA (42.6%). However, the difference in drug dose intake was not statistically significant among this polymorphisms (P> 0.124, t-test). Moreover, the one-way ANOVA test showed a significant relationship between CYP2C9*1 and drug dose(P>0.021) but no significant association was found between CYP2C9*2, CYP2C9*3 genotypes and drug dose (Figure 2).

**Table 3-A:** Frequencies of VKORC1 and CYP2C9 polymorphisms in the study population

**VKORC1 genotype**	**No. (%) patient**	**CYP2C9 genotype**	**No. (%) patient**
**GG**	34 (29.6)	*1/*1	71 (61.7)
		*1/*2	23 (20.0)
**GA**	66 (57.4)	*1/*3	17 (14.8)
**AA**	15 (13.0)	*2/*3	3 (2.6)
		*3/*3	1 (0.9)

**Table 3-B:** Frequencies of the combined polymorphisms of two genotypes of the study population

**Polymorphisms**	**No. (%)patient**	**Polymorphisms**	**No. (%)patient**
CYP2C9 *1/1 - VKORC1 G/G	22 (19.1)	CYP2C9 *1/3 - VKORC1 G/G	6 (5.2)
CYP2C9 *1/1 - VKORC1 G/A	39 (33.9)	CYP2C9 *1/3 - VKORC1 G/A	7 (6.1)
CYP2C9 *1/1 - VKORC1 A/A	10 (8.7)	CYP2C9 *1/3 - VKORC1 A/A	4 (3.5)
CYP2C9 *1/2 - VKORC1 G/G	5 (4.3)	CYP2C9 *2/3 - VKORC1 G/G	1 (0.9)
CYP2C9 *1/2 - VKORC1 G/A	17 (14.8)	CYP2C9 *2/3 - VKORC1 G/A	2 (1.7)
CYP2C9 *1/2 - VKORC1 A/A	1 (0.9)	CYP2C9 *3/3 - VKORC1 G/A	1 (0.9)

## Discussion

 In this study, the effects of CYP2C9 and VKORC1 polymorphisms on warfarin dosage in 115 Iranian patients were studied. According to our results, some of the patients can be characterised as warfarin-sensitive cases, which is currently known as a demanding issue due to development tendency of cardiovascular disorders as well as some difficulties in early phase of warfarin usage. Using the accurate does of anticoagulants is known as an important issue for responding to drugs. Among anticoagulant medicines, warfarin is widely prescribed to prevent coagulation in cardiovascular and thromboembolic disorders, reportedly.^7^^,^^14^ Therefore, it can be understood that due to the variations in individual response to warfarin, same dosage of warfarin is not suitable and curable treatment for all patients who are under anticoagulant therapy. Target international normalized ratio-prothrombin Time (INR-PT) is an important aspect that can be introduced as a significant indicator in warfarin therapy. The range maintenance between 2 and 3.5 is considered as normal range for this factor in people who are taking medications including warfarin irrespective of genotypes.^6^^,^^15^^,^^16^ Consequently, the INR test is applied to achieve the optimum therapeutic dosage of warfarin. According to previous studies, target range of international normalized ration (INR) for patients initiating warfarin therapy is between 2 to 3.5. It is estimated that via analysing the genetic location of CYP2C9 and VKORC1, the relationship between some main factors such as primary warfarin dose, steady-state dose, alterations in INR and allelic variation can be demonstrated.^17^ According to previous studies and this report, a connection between steady-state warfarin dose and CYP2C9 and VKORC1 allelic variance has been reconfirmed. Sensitivity and resistance to warfarin can be defined in a way that CYP2C9*2, CYP2C9*3 and VKORC1 polymorphisms are common in cases who are sensitive to warfarin and so require lower doses of warfarin. Additionally, those who are resistant to warfarin and therefore need higher doses of warfarin can be identified via VKORC1 missense mutation.^18^ In general sensitivity to warfarin can be defined based on clinical and laboratory parameters.^19^ Clinical definition is implied for those in whom the usual dose of warfarin is 5-15 mg per day. However, even seven days after stopping warfarin or three days after receiving vitamin K1 (3 mg per day) the INR does not dropped below 4 mg.^20^ On the other hand, the laboratory definition for sensitive warfarin patients is used in the case of those who should take warfarin approximately 10.5 mg per week until the INR blood remains in the normal range.   4^,^^11^^,^^19^  Consequently, patients with the higher dose of warfarin, required to maintain the INR in the therapeutic range, are categorized as resistant to warfarin.^5^^,^^16^^,^^19^ According to our study, the pharmacogenomics events in patients, can determine warfarin resistance or intolerance. For instance, mutation in CYP2C9 resulted in warfarin sensitivity. The product of this gene, which is expressed in a liver, is required for oxidation of a number of medications, including warfarin.   ^14^^,^^21^  After warfarin treatment, in approximately 12% of patients, severe bleeding side effects and in rare cases (approximately 2%) death occurs.^22^ Based on a case study conducted by Greaved et al*.*^16^ to determine the effects of VKORC1 (1173 C>T) polymorphisms on dose requirement and risk of severe bleeding, the intensity of the dose required to achieve the goal INR is greatly affected by presence of even one allele T in gene which can resulted in an increased risk of bleeding, as well as their sensitivity to coumarin.   6^,^^16^  Our results cover the already known relationship between CYP2C9 genotype and daily warfarin dose. There were significant differences in the mean dose requirements between each of the variant alleles compared with the wild type. Unfortunately, there was only one patient with the genotype *3/*3, despite targeting low-dose patients. Therefore, the predicted doses for this genotype should be viewed with caution. We also confirm that carriers of the VKORC1 (_1639) AA genotype require a significantly lower daily dose of warfarin than those carrying the GA or the GG genotypes. Although the VKORC1 gene has only recently been identified, a range of polymorphisms within this gene have now been described. However, in the study of Ghadam and her colleagues,^21^ warfarin resistance in an Iranian patient was found who received more than 100 mg of warfarin per day in which the warfarin concentration in plasma was much higher than the therapeutic level (approximately 22.8). However, no mutation was found in the VKORC1 gene of this patient. According to this study, no mutation was found in the VKORC1 gene of this patient. It can be realized that resistance to warfarin in these patients may be attributed to the additional variant of gene polymorphism.^19^^,^^21^ An interesting point is that although the VCORC1 gene plays a critical role in sensitivity and resistance to warfarin in eastern countries, such as Taiwan, India, Indonesia, Philippines, Thailand, Vietnam and China, different haplotype frequencies are presented among them.   5^,^^12^  The frequency of GG and AA genotype at position of-1639 of the gene in all populations studied are 50% and 21%, respectively. The abundant populations for that polymorphism in China and India are 67% and 6%, 7% and 80%, respectively. Consequently, due to the genomic individual differences, prescription of warfarin, even for people in a same geographical area needs more consideration.   3^,^^11^ 

## CONCLUSION

 The current investigation showed a significant relationship between the VCORC1/ CYP2C9 polymorphisms and warfarin sensitivity and severe side effects in Iranian patients. There are few researches that have determined the occurrence of mutations in the mentioned genes in Iranian population. The low rate of CYP2C9*2, CYP2C9*3 and VKORC1 allelic variants in this study makes it difficult to assume a precise and accurate conclusion between the warfarin dose requirements and variant gen polymorphisms. Despite achieving a statistically significant result in identification of different polymorphisms, more investigations need to be conducted on the effect of gene variants in medication usage among patients, as identification of these variants and their affects may be used as an effective factor on dose estimating of the anticoagulants. Future work may be involved in focusing on wild-genomic groups in relation to sensitivity or resistance to warfarin.
